# Attachment Theory and Maternal Drug Addiction: The Contribution to Parenting Interventions

**DOI:** 10.3389/fpsyt.2016.00152

**Published:** 2016-08-30

**Authors:** Micol Parolin, Alessandra Simonelli

**Affiliations:** ^1^Department of Developmental and Social Psychology, University of Padua, Padua, Italy

**Keywords:** attachment theory, interventions, drug-abusing mothers, mother–infant attachment, parenting

## Abstract

Children’s emotional and relational development can be negatively influenced by maternal substance abuse, particularly through a dysfunctional caregiving environment. Attachment Theory offers a privileged framework to analyze how drug addiction can affect the quality of adult attachment style, parenting attitudes and behaviors toward the child, and how it can have a detrimental effect on the co-construction of the attachment bond by the mother and the infant. Several studies, as a matter of fact, have identified a prevalence of insecure patterns among drug-abusing mothers and their children. Many interventions for mothers with Substance Use Disorders have focused on enhancing parental skills, but they have often overlooked the emotional and relational features of the mother–infant bond. Instead, in recent years, a number of protocols have been developed in order to strengthen the relationship between drug-abusing mothers and their children, drawing lessons from Attachment Theory. The present study reviews the literature on the adult and infant attachment style in the context of drug addiction, describing currently available treatment programs that address parenting and specifically focus on the mother–infant bond, relying on Attachment Theory.

## Introduction

Maternal substance abuse is a long-standing and still ongoing significant problem that establishes high levels of public health concern, given its association with negative outcomes for both the mother and her child; in fact, it is the most common psychosocial risk factor for referral to child protective services (1–4).

Recent data report a substantial increase of substance abuse among reproductive age and pregnant women (5–7), for a variety of drugs (8). It is estimated that over 90% of female opioid users are of childbearing age (7, 9, 10) and 4.5% of pregnant women use illicit drugs (7, 11). Every year, in Europe, between 6.5 and 11% of women with substance abuse problems become pregnant or give birth to a baby (12). Consequently to the increase of maternal substance abuse at delivery, the incidence of infants born to drug-dependent mothers has also augmented significantly (13–15), affecting 400,000 pregnancies annually in the United States (16, 17) and 30,000 in Europe (12).

A vast body of preclinical research indicates that different drugs affect negatively maternal care; this has been demonstrated for alcohol (18), cocaine (19), opioids (20), and ecstasy (21). Similarly, human studies strongly demonstrate that substance use disorders (SUDs) are clinical conditions that are able to interfere with relational competences (22) and parenting (23–27), affecting the establishment of the bond with the infant, with repercussions for his/her future affective-relational development.

Increasing preclinical and human studies have investigated the neurobiological pathways through which drug use can compromise parenting; data indicate that addiction mechanisms involve the same neural circuitries responsible for the initiation and expression of maternal behavior cues (28). Specifically, addiction is associated to a dysregulation of the balance between reward and stress neurobiological systems, which both undergo significant changes during the transition to parenthood. Drug exposure can affect these neurocircuits, causing a reduced sensitivity to rewarding values of infant’s cues, which are stressful instead. Thus, alterations in maternal behavior may be attributable to these differences in the reward/stressful salience of offspring’s signals.

Attachment Theory (29–31) constitutes one of the most accredited models for the conceptualization of early relationships between child and the caregiver. Bowlby postulated the universal need for intimate relationships and conceived attachment as an innate motivational–behavioral control system promoting proximity-seeking between infant and caregiver and, in turn, a feeling of security. The quality of the attachment relationship presents individual differences, which originate from the history of repeated interactions between the child and the adult; individual differences are described as secure or insecure, referring not only to infants’ manifest behavior but to the perceived availability of the caregiver too (32). Secure children experienced their caregivers’ availability, sensitivity, and responsivity, while insecurity originates form unpredictable, rejecting, or unresponsive care. Bowlby postulated that the child develops, out of dyadic experiences, complementary Internal Working Models of the attachment figures and the self, which consist of a set of expectations that guide behaviors and interpretations in subsequent relationships. Individual differences in attachment show a considerable predictive capacity for later developmental outcomes, also in adulthood, providing the prototype for later close relationships (32).

One of the applications of Attachment Theory concerns the study of the quality of attachment in groups of children with parents who belong to clinical and/or at-risk populations. The aim of this study is to evaluate the impact of parent’s pathology on the style of child-care, the quality of the mother–infant bond, and developmental outcomes in terms of attachment, as well as of general adjustment. As a consequence, various models of interventions based on Attachment Theory have been ideated, with a therapeutic purpose (namely reconstructing dysfunctional models of adult–child attachment) or a prevention intent, promoting a future infant’s developmental trajectories.

The present study illustrates the characteristics of parenting attitudes and behaviors in drug-abusing mothers. Secondly, it reviews the currently available studies based on Attachment Theory investigating adult and infant attachment styles in dyads affected by maternal drug addiction. Moreover, it illustrates the interventions on parenting commonly offered for these women and, finally, it reviews the research on programs grounded on Attachment Theory for drug-abusing mothers, their child, and the relationship they establish. Two main strategies were used to identify the studies. First, a computer-based search of the available articles was performed using relevant on-line bibliographic databases: PsycInfo, PubMed, Web of Science, EMBASE, and Cochrane Central Register of Controlled Trials. The following key terms were searched in combination as the key, title, abstract, and subject heading: mother, women, children, infant, toddler, drug, substance, addiction, abuse, misuse, disorder, attachment, intervention, program, and treatment. The search was limited to articles published in reviewed journals between 1970 and September 2015. Second, all the titles and abstracts of the produced citations were screened according to relevance, and the reference lists of the collected articles were examined to seek out potentially relevant manuscripts. The resulting papers were critically studied to determine whether they met the following inclusion criteria: the participants included mothers who were pregnant or parenting and had substance abuse problems, and/or infant–children (not adolescents or adults) of drug using mothers; studies on intervention including at least one quantitative measure of parenting, parent–infant interaction, or child characteristics.

## Attitudes and Behaviors of Drug-Abusing Mothers’ Parenting

Dysfunctional features of drug-using women’s parental role may include the tendency to ignore their child, negative affectivity dominated by feelings of anger (33), and ambivalent and incoherent attitudes (34). The parenting style can be shaped by rigidity and authoritarianism and characterized by low tolerance and the use of physical, punitive, and threatening disciplinary methods; this attitude may appear in alternation with passivity, permissiveness, and lack of supervision and control (35–38). The maternal attitude is also defined in terms of over-involvement (39), together with a tendency to isolate and refuse external influences, associated with intrusiveness and acceleration in the child’s autonomy. The need to satisfy adult’s expectancies and desires and the parental inability to fully play the parental role and function exposes drug-abusing women’s children to the risk of reversing their roles (40, 41). Limited knowledge of adequate care practices, wrong conceptions on both the pre- and postnatal developmental effects of exposure to drugs as well as on the normal developmental problems and needs contribute to the inadequacy of parental behaviors (41, 42). These mothers are at risk of ignoring important emotional aspects of the relationship with their child, increasing the likelihood of later child maladjustment (43).

In many cases, drug-using mothers’ parenting encompasses maltreatment and abusive behaviors (44) and neglect is the most widespread form (45): between 43 and 79% of children victims of maltreatment have at least one parent with addiction problems (38), and mothers who abuse substances are two or three times more at risk of maltreating their children (45) or losing custody (46). The association between substance abuse and maltreatment expresses itself both directly through behaviors acted out under the effects of substances and correlated to their research and indirectly due to irritable moods or feelings of shame and blame; an inverse pathway must also be added, for which activating abusive or maltreating behaviors lead to the seeking of substances (45).

Finally, these children are subjected to a strong discontinuity of parental care, and they deal with repetitive separations owing to abandonments that follow relapse into substances, recoveries, and incarcerations, and to a general mother’s difficulty in guaranteeing herself as a constant and available presence, both physically and psychologically (33, 38, 47). Separations from the primary caregiver are often premature, and they happen in almost one-third of the cases, during the first 2 years (44), a crucial period for the establishment of the mother–child relationship (48).

For a long time, studies have perpetuated the idea that motherhood and drug use would be incompatible (49), and the stigmatization of drug-dependent mothers have contributed to increase their feelings of inadequacy and guilt and poor parenting self-efficacy (38, 40). By contrast, empirical evidence attests the drug addiction does not fully or always compromise parenting, and a healthy caregiving relationship can be preserved despite the psychopathological condition; in fact, addiction, as other disorders, and parenting are two partially independent domains and each one can be attributable to a host of factors. Several studies highlight the lack of association between substance use and poor parent–child interactive style (50–52). Other research demonstrates that substance-using women are well aware of the potential harmful effect of their drug-related attitudes and behaviors toward the child (40, 53, 54) and they try to avoid them, while trying to meet child’s basic needs (40, 54, 55). As a matter of fact, pregnancy and motherhood may act as motives to seek treatment (56).

Noteworthy, maternal behavior can be altered by numerous other factors, such as maternal psychiatric symptomatology (36, 43) and high levels of environmental stress (57, 58), which commonly coexist with addiction, rather than by addiction *per se*.

## Attachment and Parenthood at Risk: Maternal Substance Addiction

Addiction has been conceived as an attachment disorder: drugs are used to compensate an alienated sense of self (59), to manage fearful and anxious mental states about self and others (60), to regulate emotions and restore comfort (61), and to find an alternative to attachment functions usually realized through relationships, as a result of attachment disruptions in infancy (62, 63).

The adoption of Attachment Theory as a perspective on parenthood at risk is based on the fact that a prevalence of insecure attachment models has been observed in populations of parents with mental health disorders. The meta-analysis by Bakermans-Kranenburg and van IJzendoorn (64) on groups at high psychosocial risk and on clinical groups, highlight that insecure and unresolved loss and/or trauma (U) attachment model appear in significantly superior proportions. Narrowing the focus onto parents, a meta-analysis (65) considering different psychopathological problems, behavioral disorders, and maltreatment issues, indicates that these parents showed high proportions of entangled/preoccupied (E) and unresolved loss and/or trauma (U) models. These attachment strategies can be interpreted as index of the difficulty in re-elaborating problematic early experiences and as a risk factors for the fulfilment of the parenting function, the establishment of the relationship with the infant and his/her affective-relational development (66).

In line with these works, in the specific case of substance misuse, the data highlight an association between substance use and attachment insecurity in typical and atypical samples of adults (64, 65, 67). This is valid both when attachment has been evaluated with the AAI (34, 39, 68–73), self-reports (74–89), and other interviews (90). A similar prevalence of the insecure models is also delineated by studies, which have exclusively focused on parents with drug use problems (91–98).

Although consistent research attests the prevalence of insecure attachment representations in adults with substance misuse, a specific association between drug-related disorders and a particular attachment style does not emerge (70, 98); indeed, some works have observed a prevalence of the anxious-preoccupied attachment style (73, 78, 81, 83, 99), while others have underlined the presence of the dismissing style (68, 71, 72, 77, 82, 90, 93). Others suggest an association with the unresolved loss and/or trauma (U) pattern (73, 92, 100).

As regards the specific case of drug-abusing mothers, attachment insecurity seems to characterize this clinical group in samples of mothers assessed with the AAI, indicating a prevalence of entangled-preoccupied models (E), when a three-way category distribution is considered, and of the unresolved style (U), when the four-way category distribution is applied (73, 92, 99, 101–103). Preoccupied and unresolved styles are associated with inadequate strategies of affective regulation (104–106), relying mainly on strategies of iper-activation (107). Conversely, another study has reported that substance-using mothers tend to adopt an avoidant narrative style, which seems to be associated with low levels of reflective functioning and to scarcely sensitive behaviors in interacting with the infant (108). Parent’s reflective functioning (109–111) has been considered as one of the fundamental characteristics underlying the ability to take care and protect the child, in the context of the intergenerational transmission of attachment (105). An insecure and unresolved attachment style in adulthood is associated to poor reflective abilities, which can interfere with parent’s ability to consider their child’s behaviors and feelings in terms of mental states (112). This seems to be the case with substance-using women (113–115) who are characterized by low reflective function abilities, which constitute a risk factor for the adult attachment state of mind, the mother–infant bond itself, and children’s psychosocial development (116). Unable to establish deep contact with their internal states, these women could not be capable, as a consequence, of becoming empathically attuned to their infants’ emotional experience, as a way of regulating their affective interaction (35) and of activating sensitive parenting behaviors (117). Indeed, parenting tasks, which require affective attunement and interpersonal connection have been demonstrated to be lacking in women with SUDs, who are better able to perform tasks involving activation and excitement, and that are more oriented toward action, rather than reflective abilities (118).

It is well-acknowledged that stress is strongly associated to addiction: on one side, it increases the vulnerability to drug use, and on the other side, chronic drug intake causes alterations in the stress systems (119), and this is particularly important for parents, given the changes in brain stress systems entailed by the transition to parenthood (28) and the high levels of stress associated to parenting in mothers with SUDs (57). Relatedly, attachment also serves a regulatory function, regulating negative emotions and stress, both in infancy and adulthood (106); thus, the reciprocal relationship between attachment and stress dysregulation in mothers with SUDs should be considered (120).

To get a broad developmental perspective on the transgenerational impact of addiction, studies on prenatal attachment allow an early insight on how substance use may interfere with the development of the maternal relationship with the baby-to-be, and, in turn, prenatal care and later attachment. Pregnant women who use licit and illicit drugs, such as methadone (121), cigarette (122), cocaine/heroin, and marijuana (123, 124), show difficulty developing an optimal maternal–fetal attachment, and affiliation to the fetus is characterized by mixed feelings of affection and guilt and discomfort.

Augmenting research on the neurobiology of attachment highlights the role of the reward circuitry, specifically mesocoticolimbic dopamine, in drug addiction, parenting, and social attachment (28). It indicates that drugs may interfere with the establishment of a healthy attachment bond, through the disruption or co-option of the same neural circuitry responsible for maternal behavior (41). Studies suggest a model of attachment according to which different attachment strategy represents different brain information processes and activation patterns. Specifically, dismissing mothers would rely primarily on temporally ordered cognitive information, while preoccupied mothers privilege intensity-based affective information, and secure individuals are able to integrate the two levels. Moreover, in response to infants’ cues, mothers show a different activation of mesocorticolimbic and nigrostriatal pathways and altered oxytocin production according to their attachment style. Thus, it is hypothesized that both insecure attachment and substance abuse may affect maternal brain and behavior (125).

In conclusion, the attachment theoretical perspective provides an important contribution to the study of parenting in the addiction field, affirming that adult’s representational world, associated with early attachment experiences, can become a prototype for other relationships in adulthood, including the parent–child bond (126).

## Attachment in Substance-Using Mothers’ Children

Maternal addiction represents a risk condition for a child’s socioemotional development, especially in terms of the caregiving context, since prenatal exposure to psychoactive substances does not seem to affect the quality of the attachment.

Various works have investigated attachment patterns in groups of children with mothers who abuse their children or who are affected by mental disorders (127–129), highlighting distributions, which characterize them from their normative peers, with a prevalence of insecure and/or disorganized patterns, evaluated by the Strange Situation Procedure [SSP; (130)]. More support for these data comes from the study by Greenberg, Speltz, and DeKleyn (131), which compares longitudinal research on different groups of children who are at high risk from parental conditions (medical risk, high social risk, with caregivers with a psychiatric diagnosis or other psychosocial adversity, such as alcoholism, substance abuse, family, or dyad conflict) and children with parents from the general population; their research reveals that the parents’ psychopathological condition can interfere with their ability to take care of infants, causing the establishment of an insecure bond between parent and child.

Bergin and McCollough’s work (132) highlights that an adult’s caregiving skills have an higher predictive power on the quality of the infant’s attachment with respect to the levels of intrauterine drug exposure, suggesting that the latter would not be capable of interfering with the establishment of a secure bond *per se*. Similarly, even though they have not identified an association between the severity of the prenatal exposure to substances and differences in these children’s attachment insecurity and disorganization, Beeghly and colleagues (133) report that insecure attachment bonds may be predicted only in cases where high levels of prenatal substance exposure co-occur with environmental risk variables (such as sociodemographic disadvantages, non-optimal care environment, lack of healthcare and social assistance). In this sense, it has been hypothesized that the affective-relational context provided by substance-using mothers does not permit the interiorization of a basic feeling of security in the child. This is due to a maternal vulnerability that appears in a double perspective: first, the quality of their state of mind with respect to early experiences of attachment (together with its links to the reflective function and emotional regulation) and second, the quality of care and protection that mothers provide for their children. It has been widely recognized that groups of children who belong to clinical or at-risk populations, evaluated by the SSP (130), present higher percentages of disorganized attachment with respect to the normative distribution (134). Maltreatment in the child-rearing environment, which is characterized by multiple risk factors with a cumulative effect, exposes children to a higher likelihood of developing insecure and disorganized attachment bonds in infancy (135). As highlighted by a recent meta-analytic study, insecurity and, especially, avoidant patterns results in being the most common attachment pattern in clinical or at-risk samples of children, in association with disorganized–disoriented classification (D) when four-way categorization is implied (67).

In literature, distributions of attachment patterns in groups of children with addicted mothers tend to present significant differences with respect to the normative population, but the results are not free from ambiguity and contradictions and require further investigations. The study by Swanson et al. (136), based on an assessment of the secure–insecure dimensions, shows that insecure patterns are common in groups of drug-abusing women’s children with rates of 68% higher than what has been attested in groups of children exposed to other maternal psychopathological disorders (55%), poverty (45%), and prematurity (39%). Instead, the percentages are equal to those reported in groups of children exposed to maternal alcoholism (65%) and lower only with respect to groups of children who are victims of maltreatment (86%). In another study (137), the majority of infants of mothers who used to drink alcohol in a moderate to heavy way prior to and during pregnancy, presented insecure attachment patterns at the SSP.

Focusing more in depth on insecurity, the research is not in agreement; some studies identify the Avoidant (A) category as the prevalent attachment pattern, observed in 50% of subjects (138), and others, the Ambivalent one (C) (139, 140); thus, a prevalent interactive model in the insecurity dimension has not been highlighted (97). On the other hand, maternal drug-dependence expresses its dysfunctionality in the establishment of the attachment bond, not only in terms of insecure or disorganized attachments but also may implicate a deep and generalized disorder of a child’s feelings of protection and security. As a matter of fact, data from the Italian National Health Service (141) attest an incidence of 50% for Attachment Disorders among this child population, disorders that originate from a pathological style of parenting and imply considerable alterations in the socioemotional development in early infancy, with repercussions also for child adjustment from a long-term perspective (142).

On the contrary, other studies are far from this position, revealing the absence of an association between prenatal–postnatal exposure to drugs and the establishment of insecure attachment strategies, with a prevalence of secure attachment patterns (B) among drug-abusing women’s offspring, similar to normative groups (133, 143, 144). This was also the case for two groups of children raised in a prison nursery by mothers who presented a history of substance abuse in 50–64% of cases (102).

Espinosa and colleagues (143) attribute, to the disorganized-disoriented (D) attachment category, the ability to discriminate groups at high risk with respect to the general population. Beeghly and colleagues (133) describe these children’s socioemotional behavior in terms of higher levels of disorganization and dismissing behavior, even if they do not reach the required levels to be categorized as established patterns. Overall, the empirical evidence indicates the Disorganized-disoriented pattern (D) as the trait that can better distinguish the attachment organization of the children of mothers with SUDs (132, 136–139, 143). This is the result of the combined effect of multiple factors, which exert their influence in a synergic way: child-care skills, prenatal exposure to drugs, the presence of the mother’s psychopathological disorders, and other social contextual variables. Even in families where both parents present alcohol-related problems, the predominant attachment category is Disorganization (D), in association with insecure attachment patterns, which are present to an extent equal to other groups of children at risk (145). When alcohol is the primary substance of abuse during pregnancy, prenatal exposure to this substance correlates with a high percentage of insecure patterns, in about 80% of children but scarcely supportive parenting styles have a role as mediator (146).

## Interventions on Parenting for Drug-Abusing Mothers

Despite the wide availability of gender-oriented treatments, the number of women and mothers referred to treatment is commonly low, particularly in the past (147); the explanations mainly refer to the hidden and under-estimated nature of the phenomenon of drug addiction and to women’s tendencies to define their drug-related problems in terms of psychological distress (instead of dependency on drugs) and to have poor expectations from treatment efficacy (147, 148). Moreover, many obstacles to treatment are reported, among which is the fear of being separated from their child, as interventions in the past did not always allow the coresidential living of a dyad (147, 149). For these reasons, a strong need for new protocols has emerged, fostering the implementation of programs with an integrated approach (150) and capable of addressing both mothers’ and children’s needs in equal measure (151). The principal innovative feature lies in the recognition of children’s role in facilitating their mother’s treatment itself (152), rather than assuming that children indirectly benefit from their parents’ treatment, as was previously argued (153). Reviews (154) and meta-analytic (155) studies have confirmed that a child’s presence constitutes an additional value to treatment, and they have proven its utility in respect to retention, abstinence, mental health, gestational outcomes, parenting, and employment; for this reason, children have been defined as “motivators”’ for treatment (156). Concerning child developmental outcomes, only one review is available, reporting a significant increase in several developmental areas (157).

Despite the inclusion of the child, many interventions for mothers with SUDs have revealed some shortcomings, because they often focus only on problems related to pregnancy (158) or they deal with the two main targets of treatment (mothers and children) through distinct interventions, with a lack of actual integration (150), which seems to be guaranteed only by programs explicitly addressing parenting (152). On the contrary, interventions in parenting are based on a change of perspective. Traditionally, substance abuse treatments were considered as a way of strengthening parental functioning, and the focus on parenting was motivated by the interest for the child’s well-being, while today parenting itself is considered a way of enhancing a mother’s recovery (159–162). This is in line with contributions from neuroscientific disciplines reporting a correspondence of cerebral circuits dedicated to parenting with those targeted by substances’ neurotoxic effects (41, 163, 164).

The interventions that have implemented a parenting component include both residential and home-based approaches. Inpatient programs enriched by a parenting approach lead to improvements for parenting custody, parenting attitudes and knowledge, parental stress, mother–child interaction, maternal self-esteem and addiction, and child behavioral and emotional functioning (165–169). Home-based programs have been demonstrated to be useful in improving a child’s behavior and well-being, parenting distress, mothers’ health during pregnancy, and in diminishing child maltreatment (170, 171). Interventions specifically addressed to parenting are mainly characterized by a psychoeducational or cognitive-behavioral approach (172–174), which considers maladaptive parenting as a lack of ability, information, or knowledge and aims at the correction of maladaptive attitudes and the learning of behavioral parenting skills; in other words, they overlook the emotional component of the mother–child relationship; as a matter of fact, they do not enhance mother–child interactions (172–174). Recently, the importance of the relational and interactive features of mother–child relationships has been taken into consideration and programs inspired by an interactive-relational perspective always get more credit. In this sense, intervention protocols based on Attachment Theory constitute an innovative chance for the treatment of women’s addiction and for supporting the quality of the mother–child relationship. They assume that attachment patterns related to early experiences are easily activated in the prenatal period by the parent and are then transferred to the mother–child relationship (175, 176), and they assert that the dyadic interactive quality is also crucial in delineating the developmental trajectory and the child’s future relational history (175).

## Interventions for Dyads with Drug Using Mothers Based on the Attachment Theory

Over the past few decades, Attachment Theory has represented a valuable framework for the ideation and implementation of a multitude of interventions in the clinical context, even specifically addressed to the developmental age, through a variety of approaches aimed at providing support for the relationship between the caregiver and child and to promote infant attachment security (177). Concerning the substance dependence field, the application of this theoretical approach to treatment has allowed the introduction of new models and methods of intervention, both addressed exclusively to the adult and to the mother–child dyad.

In general, treatments for addiction grounded in Attachment Theory focus on the therapeutic relationship, with its characteristics of empathy and transformative potential (61). The first and fundamental therapeutic task is *“to attach”* patients to treatment, offering primarily the possibility of new interactive-relational experiences with a sensitive and responding adult (178–180). In these terms, the bond between patient and therapist is defined as the principal vehicle for change. The therapist functions as a new attachment figure able to enhance the creation of substitutive internal working models and to provide a secure base (181). The principal element of the therapeutic relationship is recognized not merely in the establishment of the relationship with the therapist (182), but it may rather be represented in terms of feelings of security and appreciation conveyed by the therapeutic context (178). This provides the possibility of experimenting a secure base and feeling comfort enough to explore traumatic past experiences, difficulties in past attachment relationships, and those conflictual issues (states of minds, memory, and representations) that have been denied or distorted for a long time, and which have contributed to substance abuse onset and development (60, 183, 184). Another therapeutic feature is the attention paid to fostering affective regulation abilities and mentalization skills, toward one’s own and others’ behaviors and inner states. Therapists are required to help patients to develop the abilities of emotional containment and regulation, leading mothers to recognize and answer to others’ behaviors adequately, their children’s ones above all (112, 117, 185). Moreover, mothers are encouraged to approach and meet their children’s physical and psychological needs, to take care of them, and to support expectations regarding their parenting role; mothers should be able to understand how their own behavior can influence their child (178). The adoption of this treatment approach decreases the likelihood of developing insecure mother–child bonds and possibly also infant attachment disorders (136, 184, 186). Moreover, given the overlap of brain pathways involved in parenting and those affected by drugs (41, 163, 164), it is assumed that helping parents to invest in the bond with their children will, in turn, lead to a decrease in the use of substances.

Evidence of the efficacy of interventions on addiction based on attachment principles have been observed for individual outpatient treatment (46, 115, 187): the positive impact on representational, reflective, sensitivity, and caregiving maternal abilities has been verified, even after the end of the intervention. Controlled studies (178, 188) have highlighted a good efficacy of group treatment for substance-abusing mothers with a relational approach, recognizing the ability of this type of treatment to improve not only maternal adjustment but mother–infant dyadic interaction as well and to a greater extent with respect to psychosocial interventions.

In addition, empirical evidence has proven the advantages of orienting the emphasis of treatment on relational processes, as suggested by Attachment Theory, even in case of residential treatment (112, 184). Residential settings informed by attachment theories and practices have also been demonstrated to be useful in enhancing mother’s awareness of the risk of transferring, to her own child, negative interactive patterns, in buffering the risk of child maltreatment, in strengthening responsivity during dyadic interactions (attention, affective quality, taking turns, and clarity of the child’s communicative signals), and in increasing the mother’s reflective function (179, 188, 189).

Nowadays, beyond a more general approach inspired by Attachment Theory in treating drug-dependent individuals and parents, more specific intervention programs are currently available; these protocols take charge of the mother–child relationship itself considering it a specific clinical entity, qualitatively different from the mother and the child considered separately. Another valuable aspect of these programs lies in the fact that they are characterized by a clinical methodology and systematic approach, which have allowed studies aimed at testing their efficacy empirically to be conducted.

One of the first program ideated is the Breaking the Cycle protocol (BTC) (190, 191). It assumes a preventive approach, and it targets the early developmental stages, from the prenatal age for the first 6 years of the child’s life. BTC adopts a comprehensive and integrative model, which coordinates different services, otherwise, fragmentarily offered (psychotherapy for parent and child, parenting intervention, developmental services, health assistance, counseling for substance addiction, and kindergarten); instead, in BTC, these treatment components are unified, and the links refer to the central value attributed to the relationship. First, the relationship is considered for its social meaning, and the program aims to contrast the isolation that these women often experience and to instill, in the women, the importance of establishing and maintaining supportive relationships. Frequently, the first contact with drug addiction services is requested by women with SUDs in order to obtain merely physical health care and for material support; only at the next moment, do they find out the possibility of finding a warm welcome and acceptance and consideration by the service operators. Furthermore, the women’s past and present relational problems, which may play an interfering role in the establishment of the mother–infant relationship, are conceptualized in terms of “relational images,” and the mothers are accompanied by an awareness of the possibility that these images can be transferred to the child. The purpose is to sustain and enhance the dyadic interaction, offering an affective and corrective alternative relational model with respect to those previously experienced, with coherence, reliability, and predictability, in accordance with Fraiberg’s teaching (192). This intervention model has been demonstrated to be effective both in prenatal and neonatal outcomes, also for long-term developmental trajectories; an increased parental competence has also been translated as a lower likelihood of separation of the child from their mother (193).

The Mother and Toddler Program (MTP) (194) is an intervention protocol addressed at substance-using women’s offspring in their pre-education years, specifically, between 12 months and 3 years. It consists of 12 weekly therapeutic session. At the base, is the need to adopt a maternal representative world and reflective function as targets of evaluation and intervention in the individual psychotherapy context, and the idea that these have important secondary effects on caregiving behavior, mental health, and substance abuse. The MTP includes an initial evaluation, conducted with different methods of assessment for multiple aspects. First, the adult representational component is investigated through attachment-based interviews such as the *Parent Development Interview – Revised* (195) and the *Working Model of the Child Interview* (196); second, observational procedures, more or less structured, of early adult–infant interactions are applied. Videos of the recorded interactions are reviewed and commented on with mothers, providing them with feedback; this therapeutic methodology allows, first of all, the promotion of maternal involvement in the relationship with her baby and, moreover, to strengthen the mother’s affective regulation and reflective function. A randomized control trial compared this program with a psychoeducative–behavioral intervention: the MTP has been demonstrated to be effective for increasing mothers’ reflective functions and the quality of their maternal representations (individuated as the main mechanisms of change) and *caregiving* behavior, in addition to offering benefits for psychological distress and abuse issues (187). Furthermore, the empirical evidence attests that the increase in maternal reflective abilities, fostered by the intervention focus on mentalization, may be translated in a child’s more adaptive regulation abilities at 2 and 3 years of age, a developmental period in which the competences of regulation constitute one of the most important developmental tasks (46, 115, 187, 197). In addition, empirical evidence was recently provided concerning the mechanism of change that has been hypothesized to be beneath this type of intervention; namely, the modifications of the reflective functioning abilities and of the quality of the maternal representations are associated with those ascertained differences in caregiving behaviors (197).

The application of attachment constructs has also been achieved in a residential treatment context, where a short but intensive protocol of intervention for new mothers, named Attachment and Biobehavioral Catch-up (ABC) (198), has been ideated for children in the charge of child-care services. Similarly to the previous ones, even this intervention program consists of sessions in which mother–child dyadic interactions are recorded and encompasses the guidance of a *“parenting coaching.”* However, ABC is characterized by some specifics: the main focus is on attachment-inspired themes, such as the reduction of frightening maternal caregiving, the enhancement of maternal sensitivity, and the ability to follow the child’s guide in the interaction; in sum, parental behaviors are considered the elective target of intervention, instead of the internal representational world. Demonstrations of efficacy in positively modifying a mother’s behavior, already verified in other settings of intervention (199), are confirmed even with mothers with substance dependence (126).

The *Tamar’s Children* protocol was originally ideated for mothers in the detention system, who, as previously highlighted, frequently presents a history of use or abuse of substances (200). It constitutes a variation of the Circle of Security program (COS) (201–203), which mainly addresses the pre-education age. The COS identifies the caregiver as the focus of treatment, considering the secure base and safe haven concepts as fundamental theorizations for treatment and using recorded dyadic interactions and therapeutic and psychoeducational discussions. *Tamar’s Children* specifically addresses the prenatal age and the first year of a child’s life, and it is part of an integrated intervention, which is also directed to other purposes such as substance abuse, the treatment of different *sequelae* associated with traumatic events, and work training. The efficacy of this program was tested by an empirical study using the SSP (130) during the 12th month of a child’s life for children previously involved in the protocol: a total proportion of insecure attachment emerged in this group (independently from the category considered), which is comparable to the one that has been observed in low-risk populations (200). This result was interpreted by the authors as evidence of the efficacy of this model of intervention in preventing the co-construction of infant insecure attachment patterns, in spite of maternal clinical characteristics.

The *Cherish the Family* program (204) was developed with the purpose of supporting drug-abusing mothers of young infants (aged 0–3), strengthening the mother–infant bond, and promoting family reunification when there is the risk of losing child custody. The program consists of comprehensive case management services, addressing parents’ health, parenting, daily life management, and it focuses on the reinforcement of the mother–baby relationship, taking advantage of the *Promoting First Relationship* curriculum (205). The curriculum is grounded in Attachment Theory, and it intends to support parenting and to foster secure relationships and child socioemotional development through the promotion of parents’ reflective abilities, in terms of empathizing with their child’s distress, understanding their child’s behaviors and signals, and thinking about their child’s developing mind. *Promoting First Relationship* has shown proof of its efficacy in ameliorating parents’ understanding of socioemotional needs, observed caregivers’ sensitivity, and toddlers’ competences in dyads with placement disruption; unfortunately, no significant change was detected in child attachment security (206). The results (204) support the power of *Cherish the Family* in increasing drug-abusing mothers’ responsiveness while interacting with their child and child well-being, but no data on infant attachment outcomes are available.

Finally, two other interventions can be included among the attachment-based programs for substance-abusing parents and children, but, unfortunately, no statistically significant results on their effectiveness have been provided. First, an eight-session group program based on the *Circle of Security* model (201–203) was delivered to eight opiate-dependent parents of children between 0 and 5 years of age; in a pilot study (207), the program showed good retention and was positively rated by the parents, and the results indicated improvements in the caregivers’ psychological well-being, including substance use, but no clinically significant change in parent–child interactions. Second, an intervention for parents with addiction problems has been designed, which uses infant massage to foster the attachment bond with their infant through the learning of an attuned and rhythmic touch, better recognition of infant internal states, and interactive non-verbal responses (208). The program, called *Fostering Mindful Attachment (FMA)*, consist of 10 classes in which educational topics, mindfulness sessions, and infant massage instructions and practice are combined; the findings show some promising trends in parenting variables and a protective role against parent–infant separation, but they are only preliminary.

## Maternal Addiction and Mother–Child Attachment: Reflections, Shortcomings, and Future Perspectives

Attachment Theory offers a promising framework for the evaluation of the quality of the mother–child bond, as well as for the treatment of maternal addiction and for supporting and promoting a child’s socioemotional well-being and attachment security. Thus, treating drug addiction and promoting secure attachment bonds are increasingly recognized as two components of psychological health care, which are systematically related. As stated by Pawl (209), dyadic interventions might offer the opportunity for mothers to reflect on their past and present emotional experiences that lead to reliance on substances and interfere with their emotional and behavioral attitudes toward the child.

The first limitation concerns the inconsistency of the results on the distribution of attachment patterns (especially the insecure ones) in groups of addicted women’s children. The absence of the prevalence of one specific attachment model may lead to the assumption of the existence of multiple factors capable of influencing the co-construction of the infant attachment bond; besides, it limits the current knowledge about the impact of maternal addiction on the establishment of the primary bond. In this sense, some shortcomings are present in studies that have investigated the intergenerational transmission of the insecure and disorganized attachment models from mother to child, but, more in general, this is quite a controversial theme in the study of this attachment. At the moment, it is not possible to give a clear picture of the processes of influence exerted by the relational and representational mother’s world on the quality of the bond with their child. Further controlled studies on larger samples may provide more specific and exhaustive indications about the effect that, in terms of attachment, a mother’s addiction has on the modalities of care and protection toward their infant, as well as on structuring their child’s primary attachment.

Simultaneously, a strong need has emerged for interventions more specifically focused on women’s addiction and able to support their motherhood and the construction of a positive mother–infant relationship. Even though different interventions aimed at improving parenting skills have been implemented for drug-addicted women and their children, attachment-focused therapy for addiction is still rare (210); moreover, the results of efficacy are not univocal, emphasizing on the one side, the need for more integrated approaches and, on the other side, the importance of focusing specifically on the relational features of mother–infant interactions. In this sense, the Attachment Theory may constitute a privileged perspective to plan and develop early interventions. As a matter of fact, not only might home-based programs be enriched by the education and training of social workers about attachment assumptions, but above all, residential treatment protocols seem to offer the ideal context in which to realize attachment-informed interventions.

## Author Contributions

MP and AS both contributed to the ideation of the design of the work, data acquisition, and interpretation. After a discussion on the main aspects of the work, they found an agreement on its final version.

## Conflict of Interest Statement

The authors declare that the research was conducted in the absence of any commercial or financial relationships that could be construed as a potential conflict of interest.
